# EVALUATING CPLA2 ACTIVITY IN APOE4 CSF AFTER PREVENTE4 DHA SUPPLEMENTATION

**DOI:** 10.1093/geroni/igae098.4173

**Published:** 2024-12-31

**Authors:** Temi Ogunade, Hussein Yassine, Marlon Vincent Duro

**Affiliations:** USC Leonard Davis School of Gerontology, Los Angeles, California, United States; USC Keck School of Medicine, Los Angeles, California, United States; USC Keck School of Medicine, Los Angeles, California, United States

## Abstract

Alzheimer’s disease (AD) is a neurodegenerative disorder characterized by cognitive decline, neuroinflammation, amyloid plaques, and neurofibrillary tangles. The apolipoprotein E ε4 (APOE4) allele is the greatest genetic risk factor for late-onset AD. Most intervention studies involving omega-3 supplements share inconsistent results in APOE4 carriers, calling for a deeper examination of the mechanisms behind its anti-inflammatory properties. Phospholipase A2 (PLA2) enzymes are involved in the hydrolysis of membrane phospholipids, releasing fatty acids like docosahexaenoic acid (DHA) and arachidonic acid (AA) which modulate intracellular signaling pathways that influence differences in inflammation and lipid metabolism similar to AD pathology. The PreventE4 trial is a randomized, double-blind study that investigates the relationship between the APOE4 genotype and brain DHA delivery. 368 cognitively unimpaired adults with cardiovascular risk factors for dementia are given high DHA supplementation or a placebo. Among the group, 184 participants are APOE4 carriers and 184 are non-APOE4 carriers. The study questions if APOE4 limits DHA delivery to the brain. Our lab has demonstrated that calcium dependent PLA2 activity (cPLA2) is increased in APOE4 carriers with AD dementia. We hypothesize that APOE4 is associated with greater cPLA2 activity in cerebrospinal fluid (CSF), resulting in a lower increase in CSF DHA levels after supplementation. I propose to measure cPLA2 activity in CSF samples from PreventE4 participants using a liposomal-based fluorescent assay. The results will provide better insight on how APOE4 complicates responses to DHA supplementation. Knowing this, we will be able to target DHA’s metabolism and identify who benefits most from omega-3 supplementation.

